# SWS: accessing SRS sites contents through Web Services

**DOI:** 10.1186/1471-2105-9-S2-S15

**Published:** 2008-03-26

**Authors:** Paolo Romano, Domenico Marra

**Affiliations:** 1 Bioinformatics, National Cancer Research Institute (IST), Genova, I-16132, Italy

## Abstract

**Background:**

Web Services and Workflow Management Systems can support creation and deployment of network systems, able to automate data analysis and retrieval processes in biomedical research. Web Services have been implemented at bioinformatics centres and workflow systems have been proposed for biological data analysis.

New databanks are often developed by taking into account these technologies, but many existing databases do not allow a programmatic access. Only a fraction of available databanks can thus be queried through programmatic interfaces. SRS is a well know indexing and search engine for biomedical databanks offering public access to many databanks and analysis tools. Unfortunately, these data are not easily and efficiently accessible through Web Services.

**Results:**

We have developed ‘SRS by WS’ (SWS), a tool that makes information available in SRS sites accessible through Web Services. Information on known sites is maintained in a database, srsdb. SWS consists in a suite of WS that can query both srsdb, for information on sites and databases, and SRS sites. SWS returns results in a text-only format and can be accessed through a WSDL compliant client. SWS enables interoperability between workflow systems and SRS implementations, by also managing access to alternative sites, in order to cope with network and maintenance problems, and selecting the most up-to-date among available systems.

**Conclusions:**

Development and implementation of Web Services, allowing to make a programmatic access to an exhaustive set of biomedical databases can significantly improve automation of in-silico analysis. SWS supports this activity by making biological databanks that are managed in public SRS sites available through a programmatic interface.

## Background

### Technologies for the automation of biological data analysis

Biological data is available in heterogeneous information systems that are distributed over the Internet. In this situation, data integration is needed in order to achieve a better and wider view of available information, to automatically carry out analysis and/or searches involving more databases and software and to perform analysis involving large data sets. In this frame, the need is felt for a system that is able to improve the information accessibility by raising it at an automatic level.

Among current ICT technologies, Workflow Management Systems (WMS), in connection with Web Services (WS), seem to be the most promising ones. A recent paper presented a methodology for the automation of *in-silico* data analysis processes through workflow management systems that takes into account the synergic use of XML schema, XML data storage, data and task ontologies, Web Services, workflows systems and enactment portals [1].

More specifically, reasons for the setting up of WS in bioinformatics have already been presented by many authors [2-4]. WS have already been implemented by many Institutes and service centres in the biomedical field. Partial lists of Web Services for bioinformatics are available at the myGrid Wiki site [5] and in the Taverna web site [6]. Also, Web Services can be retrieved and accessed through the MOBY Central [7], a WS archive based on BioMOBY [8], an open source software that implements an architecture for the discovery and distribution of biological data through Web Services.

Workflows are defined as “computerized facilitations or automations of a business process, in whole or part” (Workflow Management Coalition, WfMC) [9]. Their goal is the implementation of data analysis processes in standardized environments and their main advantages relate to effectiveness, reproducibility, reusability of procedures and of intermediate results and traceability.

Some WMS have also been proposed in bioinformatics [10-18], the Taverna Workbench [19,20] from the European Bioinformatics Institute (EBI, [21]) probably being the best known among open source applications developed by public research institutes. It is able to build complex analysis workflows, to access both remote and local processors of various kinds, to launch execution of workflows and to display different types of results, including text, web pages and various kinds of images and diagrams. Processors that can be used through the Taverna Workbench include Web Services. One kind of Web Services that can be accessed by using Taverna Workbench, are those implemented by using Soaplab [22,23], a tool for the rapid deployment of Web Services developed at EBI by Martin Senger.

### Extending available contents

New information sources are often developed by taking into account above mentioned technologies. Otherwise, many databases, developed during previous years, do not offer a programmatic access to them: this reduces the information that is available for automated analysis.

Sequence Retrieval System (SRS, [24,25]) is a well known indexing and search engine for biomedical databanks, developed by Thure Etzold at EBI and currently distributed by BioWisdom Ltd. SRS is able to efficiently query a set of local databases. Among its most original and useful features are the possibility of querying many databases together and of integrating data retrieval and data analysis in the same tool. SRS is usually searched through its user interface, the CGI compliant software wgetz, that offers a rich set of alternatives ways to interact with SRS and to compose queries in its query language. It is possible to interact with SRS by creating *ad hoc* URLs that either specify a query or request a specific output, such as the list of available databanks and the description of fields composing a library. Examples of this are presented in table 1. From its version 8, SRS is based on a new architecture where software components communicate among them by using Web Services. These do not follow agreed W3C standards, are proprietary and are not publicly available. For these reasons, they cannot be used in a open environment for integrating contents of SRS libraries with other network data sources.

**Table 1:** Some ways to interact with SRS 7 and to compose queries in its query language.

Show list of databanks	wgetz?-page+databanks
Show databank's information	wgetz?-page+LibInfo+-lib+***libname***
Show databank field's information	wgetz?-page+FieldInfo+-lib+***libname***+-bf+***fieldname***
Query databank	wgetz?-e+[***libname***-***fieldname***:***terms***]
Query multiple databanks	wgetz?-e+[{***libnames****}*-***fieldname***:***terms***]
Query databank (return text only)	wgetz?-e+[***libname***-***fieldname***:***terms***]+-ascii
Query databank (return some fields only)	wgetz?-f+***fields***+[***libname***-***fieldname***:***terms***]+-ascii

This table shows some of the many possibilities of interacting with SRS version 7 by submitting arguments and options to wgetz through the URL (HTML forms GET method). This kind of interaction is the basis of perl scripts that are run by SWS services.

SRS used to be free for academic and no-profit institutes. Through its many public sites, it offers access to more than 1,000 databanks and about 180 analysis tools. The most important databank, like GenBank, Interpro, and Gene Ontology, can be included in many sites. This partial redundancy can be used to overcome possible sites' crashes and network faults that can occur at any time. Although some SRS libraries are subsets or subsections of other, this does not mean that they have less relevance and interest for researchers. For the sake of the SRS system they effectively are different databases, each of them having its special interest. Advantages of considering such libraries as real databases include, for subsets, improvement of performances, and, for subsections and views, simplified data management. The latter topic is especially important when considering automated analysis. Unfortunately, SRS libraries are not accessible through WS. So, a tool that would allow to interact with these databanks through Web Services would be extremely useful.

### Previous work for Web Services for SRS libraries

To our knowledge, the only attempt to develop a system that is able to interact with SRS through Web Services was made by the group of Prof. Douglas Kell at the University of Manchester [26]. They developed srs2acd, a set of java classes that are able to create AJAX Command Definition (ACD) [27] files for accessing each databank that is available in an SRS site, given its main URL. These files can then be used for deploying Web Services by using the Soaplab tool. Each one of the resulting Web Services is then specially devoted to a unique implementation, i.e. to one database in one SRS site. Main limitations of this tool are that each implementation has a related Web Service, thus producing a high number of services, and that access to alternative, but equivalent, WS is not automatically managed.

### The list of public SRS sites

A list of SRS public sites [28] is maintained by BioWisdom Ltd [29]. This information is available as a simple, partially structured, HTML page. The list allows to identify all available copies of a given database and to compare them on the basis of their number of entries. It also provides the status of each site at a given date, although it is only updated once per day.

As previously said, the same library can be included in many SRS sites. This duplication and partial overlap can support fault tolerance and help overcome sites' crashes and network faults. An analysis of the status of sites (active and non-active) over a period of 65 consecutive days showed that less than the 50% of sites was always active in that period.

### Transparent access to SRS contents

A system for accessing SRS contents, without the need of specifying which implementation should be queried, would be useful. This ‘transparent’ access to SRS would allow researchers using workflow management systems to abstract their workflow from the connection details and would offer them a way for avoiding network problems. Such system should be able to check which sites are available at the time of execution of the workflow and select the ‘best’ site, i.e. the site including the most up-to-date information, among active sites.

In this paper, we present a set of Web Services that makes information available in public SRS sites accessible as a whole, not singularly, through a programmatic access. It enables WMS to access all active SRS sites and to query needed libraries. It also manages access to alternative, but equivalent implementations, by also selecting the most up-to-date among available systems.

## Results

### A suite of Web Services

We have developed SWS (SRS by WS), a suite of WS allowing to query biological databases available in public SRS sites, without specifying which one, and to return results in a simple text-only format. It allows to check sites, to query selected libraries and to retrieve essential information on sites, such as lists of included databases and tools, and on databases, such as sites where they are implemented and related sizes/versions.

SWS can be invoked by specifying the name of the databank to be queried and the query terms. It then automatically choose the best site, performs the query and returns complete results. Users can also specify the following information: the SRS site to be queried, the fields where the information must be searched, the desired output fields.

### Available Web Services

SWS currently includes five Web Services. The following three WS allow to retrieve information on available databases. getDBs retrieves acronyms of all libraries that are available in a specified site, or in all known sites, if none is specified. Similarly, getSites retrieves acronyms of all SRS sites that include a specified library, or of all sites if no library is specified. Finally, getImplementations retrieves all implementations of a specified library. These WS are only available for informative reasons, they do not actually query any SRS site. Instead, results can be used to identify available libraries and to choose sites and libraries to be queried in following steps.

The fourth WS, querySWS, allows to actually perform queries on a specified library. The query (i.e. terms that must be searched in the database) is a mandatory parameter. The site, instead, can be omitted. In this case, SWS identifies the best one by selecting, among those that are active, the site where that specific library has the greatest number of entries and, when more sites have the same number, the most recent version of SRS (this function is currently limited to SRS versions 6 and 7). Further parameters of this WS allow to determine which parts (fields) of the library must be queried, and which parts of the entries (records) must be returned.

Finally, the fifth WS, testSites, allows to check for the availability of a site at a given time. Input parameters are the acronym of the site to be checked, the number of retries (in case of initial failure) and the time between retries. All these parameters are optional. When no site is specified, all sites are tested.

WS name, inputs and outputs are summarized in table 2.

**Table 2:** SWS Web Services and related inputs and output.

**Web Service name and description**	**Inputs**	**Outputs**
**getDBs** Retrieve data from srsdb about libraries.	**lib:** the acronym of the library. Default value: ALL	**Output:** list of libraries' acronyms
**getSites** Retrieve data from srsdb about SRS sites.	**site:** the acronym of the site. Default value: ALL	**Output**: list of sites and related info
**getImplementations** Retrieve data from srsdb about implementations of libraries in SRS public sites.	**lib:** the acronym of the library. Default value: ALL **site:** the acronym of the site. Default value: ALL	**Output**: list of implementations and related info
**testSites** Check actual availability of sites.	**site:** the acronym of the site. Default value: ALL **retries:** the number of retries after an initial failure. Default value: 1 **time:** time between retries. Default value: 5 secs	**activity:** status of sites in one of the forms: <site> is active<site> is not active
**querySWS** Query a specified library and return entries related to queried terms.	**lib:** the acronym of the library. No default value. **site:** the acronym of the site. Default value: best site **query:** the query (terms to be queried). No default value. **in_fields:** list of library's fields to be searched. Default: AllText **out_fields:** list of library's fields to be returned. Default: all	**Entries**: library's entries matching query

This table lists SWS services by specifying their name, function, input and output.

### Availability of the tool

SWS is available on-line [30]. Through this site, users and software agents can retrieve WSDL descriptions of Web Services included in SWS. Some WSDL compliant WMS, such as Taverna Workbench, can directly interact with SWS through this interface and execute the services.

A support site for SWS is also available on-line [31]. In this site, a description of the software and usage information of single WS are provided, together with contacts data. We plan to allow, in the near future, downloading all files needed to implement a local version of SWS, including data structure, scripts for retrieving information on available sites, ACD definitions for a Soaplab implementation of Web Services, scripts that actually implement the services and installation instructions. SWS is being developed as an open source and it is under refinement and continuous development.

## Discussion

We believe automation of data analysis and retrieval processes will offer bioinformatics the possibility of implementing a really machine-oriented, distributed analysis environment. For this to happen, the development and implementation of WS that allow to make access to an exhaustive set of biomedical databases and analysis software is needed. Only a few of the many biological data sources that are currently available on-line can be queried through standard programmatic interfaces. Our tool SWS contributes to overcoming this limitation by supporting programmatic access to known SRS sites. Contrary to other similar tools, SWS offers a unique point of access for all sites because it leverages from a database of available sites, databanks and implementations that is derived by BioWisdom's list of public SRS sites and kept up-to-date by using own scripts. BioWisdom's list is a useful reference enabling SWS not to start from scratch. It is used both as a starting list and to check for new sites. An exhaustive list of public SRS sites is very difficult to achieve, we hope that in the near future we can set up an alternative list and receive information on new sites. By querying this database, SWS can determine which is the best site for each databank at any given time, and it therefore overcomes, at least partially, possible problems arising from sites' crashes and network faults. This is achieved b using the number of records included in the databank since, usually, this increases each time a new version of the databank is released. Information regarding databases' version is not usually available and, thus, it can only rarely be used. SWS also allows for the creation of workflows that are not dependent on one single site and can, therefore, work more consistently.

SWS currently presents some limitations that we plan to overcome in the near future. It is currently not able to query sites using SRS 8, due to the difficulty in building queries through wgetz with this version. This limitation can be considered a minor one, since the vast majority of public SRS sites is still based on SRS 7. We plan to overcome this limitation by collaborating with administrators of SRS sites with specific expertise on this version. Another limitation of SWS is that it can presently only be efficiently used when the SRS configuration of a databank is known. In this case, both searched and retrieved fields can be specified. This can be overcome by adding support for the retrieval of descriptions of databanks fields. Another limitation refers to the scarce information that SWS reports about the databanks and sites it used for a query. In fact, it's true that users are not informed about which data set was used. This problem is going to be faced by including detailed information on data provenance, mainly comprising date, time, site, database, db version, number of records. Finally, access to tools (analysis software) that are available in SRS sites is not possible. We don't see this point as a limitation, because of the availability of alternative Web Services offering access to this software, like, e.g., EMBOSS [32] related ones.

## Conclusions

Web Services are the most promising among ICT tools, in view of the automation of network based data retrieval and analysis in biology. We developed SWS, a suite of Web Services that support query and retrieve of data from databases included in public SRS sites. These Web Services can increase the amount of data that currently is available for the setting up of complex workflows and can in fact improve automation of *in-silico* analysis by extending possible applications.

SWS is available for interested researchers through their workflow management systems, provided they are SOAP compliant and can use WSDL descriptions. The tool will soon be available for downloading from the SWS support site for local implementations.

SWS is currently being further developed in the sphere of the Laboratory of Interdisciplinary Technologies in Bioinformatics – LITBIO [33].

## Methods

### The list of public SRS sites

The list of public SRS sites that is maintained by BioWisdom Ltd is divided into three sections. The first section is made up of a list of sites. The second includes libraries (i.e., databases) and related implementations (i.e. sites where the library is available). The third section includes tools (i.e., analysis software) and related implementations. BioWisdom's list is very essential, and it only includes a few data. See table 3 for a commented list of available data.

**Table 3:** Information published in BioWisdom list of public SRS sites.

**Information on the sites**	
Site's acronym	Short acronym of the site, often the acronym of the institute where it resides
Site's description	Long description of the site, usually the name and country of the Institute hosting the site
Site's URL	The URL for the home page of the SRS site (not the institute)
No of libraries and tools at the site	The number of libraries and the number of tools that are available at the site. Sites that are not currently active are labelled here with ‘Could not access site’
SRS version of the site	The number of the version of SRS that is available at the site. It ranges from 6 to 8. The most frequent version is 7.1.x .
**Information on the library**	
Library's acronym	Short acronym of the library, usually an internationally known acronym
**Information on the implementation of a library in a site**	
Site	Acronym of site, same as above
URL	The URL of the CGI program (either wgetz or srs, depending on SRS version). Specific parameters and options are appended to this URL to compose the query.
No of entries	Number of entries of the library in the current site
**Information on the implementation of a tool in a site**	
Site	Acronym of the site, same as above
URL	The URL of the CGI program, same as above for libraries

This table lists and comments information that is included in the list of SRS sites that BioWisdom maintains. This information is included in a HTML file having a regular disposition of tags, allowing to extract data by automatically scanning the file.

The list allows to identify all available copies of a given database. Unluckily, not all SRS sites include information on databases' version, so this data cannot be regularly used. In order to compare databanks, the number of their entries can be used instead. Usually, this number depends on the version of the database and it is higher for later releases. So, one can assume that the higher is the number of entries of a database, the most recent is the release.

### The local database srsdb

**Figure 1:**
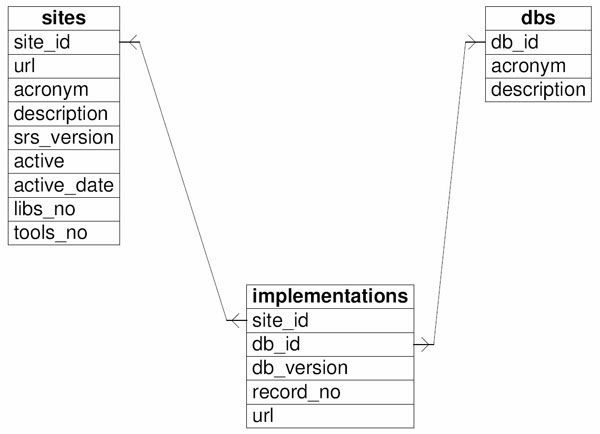
**Schema of the srsdb database.** This figure shows the schema of the srsdb database. Srsdb includes three tables: one for sites, one for libraries and one for implementations. The two former tables describes sites and libraries as separate entities, the latter table is a join that specifies characteristics of implementations of specific libraries in specific sites.

BioWisdom's reference list is checked daily and information regarding available sites and libraries is stored into a local database, srsdb, based on MySQL open source database management system [34]. Srsdb contents are kept up-to-date by running a perl script that checks the actual status of the sites each hour. Srsdb includes three tables, one for sites, one for libraries and one for implementations. While the two former tables are used for describing the sites and the libraries as separate entities, the latter table joins them by specifying characteristics of the implementations of each library in every site. The information included in the database essentially is the same that is included in the list of public sites. Only a few data are retrieved by further querying the sites. Figure 1 shows the simple srsdb schema.

### Web Services

Web Services were implemented by means of perl scripts. These can also be run through command lines. Two types of services were implemented, one for querying srsdb and one for querying and checking SRS sites. Services and command lines usage information are available in additional file 1.

The first three scripts, i.e. getDBs, getSites and getImplementations, just interpret line arguments, query the srsdb database and return results to standard output. The fourth script, querySWS, initially queries the local database for retrieving information on available sites and databases that is needed to perform the remote query and then it carries out the actual request by searching the chosen site. Results are retrieved by using the GNU Wget non-interactive network downloader and returned to standard output, after checking for errors. The last script, testSites, retrieves from srsdb information on sites to be checked and then check if they are active, returning this information to the user.

### Web Services deployment

Web Services have been deployed by using Soaplab [22,23], a SOAP-based Analysis Web Service tool providing a programmatic access to local, command-line applications and to the contents of ordinary web pages. The only requirements of Soaplab are the Apache Tomcat servlet engine [35] with the Axis SOAP toolkit [36], a Java Virtual Machine [37] and, optionally, perl [38] and MySQL. Once the server has been installed, new Web Services are deployed (added to the system) by defining simple descriptions of related execution commands. Definitions are written in the AJAX Command Definition (ACD) language [27] and are then converted into XML before they can be used by remote users.

## List of abbreviations used

ACD: AJAX Command Definition

EBI: European Bioinformatics Institute

EMBOSS: European Molecular Biology Open Software Suite

HTML: HyperText Markup Language

ICT: Information and Communication Technology

LITBIO: Laboratory of Interdisciplinary Technologies in Bioinformatics

SOAP: Simple Object Access Protocol

SRS: Sequence Retrieval System

SQL: Structured Query Language

SWS: SRS by Web Services

URL: Uniform Resource Locator

W3C: World Wide Web Consortium

WfMC: Workflow Management Coalition

WMS: Workflow Management System

WS: Web Services

WSDL: Web Services Description Language

XML: Extensible Markup Language

## Competing interests

The authors declare that they have no competing interests.

## Authors' contributions

PR conceived the study, participated in its design, coordinated and contributed to the development and implementation of software, and drafted the manuscript. DM participated in the design of the study and contributed to the development of software. All authors read and approved the final manuscript.

## Supplementary Material

Additional file 1 SWS: usage of scripts
Description: SWS perl scripts and their usage are presented in the classical “usage style” where the script name is followed by all possible parameters, values and defaults. Click here for file
