# Supplementary material for: SWS: accessing SRS sites contents through Web Services
Source: BMC Bioinformatics. 2008 Mar 26;9(Suppl 2):S15. doi: 10.1186/1471-2105-9-S2-S15 (PMC2323664; doi:10.1186/1471-2105-9-S2-S15)
Supplement: Additional file 1 — SWS: usage of scripts Description: SWS perl scripts and their usage are presented in the classical “usage style” where the script name is followed by all possible parameters, values and defaults. [file 1471-2105-9-S2-S15-S1.pdf]

SWS: usage of scripts

SWS perl scripts are command line programs that are launched by the Soaplab tool upon request of the execution of a Web Service by a remote user. There is a straight one-to-one correspondence between Web Services and scripts, where Web Services I/O correspond to parameters and results of scripts.

In this document, scripts and their usage are presented in the classical "usage style" where the script name is followed by all possible parameters. All parameters include a name and a value. The name is preceded by a minus sign. The value is shown as a tagged name, i.e. it is enclosed between a 'less then' and a 'greater then' operators, meaning that it must be substituted by an actual value or string. Value types and default values are also shown. Optional parameters are enclosed within square brackets. The usage text is followed real examples.

1. getDBs

Retrieve data from srsdb about libraries.

Usage: getDBs [-lib <db_acronym>]

Optional parameter <db_acronym> is the acronym of the library, as used in the BioWisdom list of public SRS sites. If it is not specified, all libraries are returned.

Examples:

getDBs

getDBs UNIPROT

getDBs 'ICLC DSMZ_MUTZ'

2. getSites

Retrieve data from srsdb about SRS sites.

Usage: getSites [-site <site_acronym>]

Optional parameter <site_acronym> is the acronym of the SRS site, as used in the BioWisdom list of public SRS sites. If it is not specified, all sites are returned.

Examples:

getSites

getSites EBI

getSites 'EBI IST'

3. getImplementations

Retrieve data from srsdb about implementations of libraries in SRS public sites.

Usage: getImplementations [-site <site_acronym>] [-lib <db_acronym>]

Optional parameters <site_acronym> and <db_acronym> respectively are the acronym of the SRS site and of the library, both as used in the BioWisdom list of public SRS sites. If each of them is not specified, all sites/libraries are returned.

Examples:

getImplementations -s EBI -l UNIPROT

getImplementations -s EBI

getImplementations -l UNIPROT

getImplementations

4. querySWS

Query a specified library and return entries related to queried terms.

Usage: querySWS -lib <db_acronym> -query <terms> [-site <site_acronym>] [-input <input_fields>] [-output <output_fields>]

Parameters are defined as follows:

<db_acronym> is the acronym of the library to be queried (mandatory),

<terms> are one or more words to be searched in the library (mandatory),

<site_acronym> is the acronym of the site to be queried (optional),

<input_fields> are one or more field's names where terms must be searched (optional),

<output_fields> are one or more field's names to be returned (optional)

Default values are as follows:

<site_acronym> does not have a default. The "best" site is chosen automatically.

<input_fields> default to AllText and <output_fields> default to all fields.

Examples:

querySWS -lib ICLC -query hela

querySWS -lib ICLC -query hela -site EBI -input_fields cell_line_name

querySWS -lib ICLC -query hela -output_fields "cell_line_name hazard"

5. testSites

Check if an SRS site is active.

Usage: testSites [-site <site_acronym>] [-retries <retries_no>] [-time <sleep_time>]

Parameters are defined as follows:

<site_acronym> is the acronym of the site to be queried (optional),

<retries_no> number of retries after failing (optional),

<sleep_time> time between retries, in seconds (optional)

Default values are as follows:

<site_acronym> default to all sites.

<retries_no> default to 1 and <time> default to 5 seconds.

Examples:

```
testSites -site EBI

testSites -site EBI -retries 10 -time 5

testSites -retries 10 -time 5

testSites
```
